# High-throughput generation of midbrain dopaminergic neuron organoids from reporter human pluripotent stem cells

**DOI:** 10.1016/j.xpro.2021.100463

**Published:** 2021-04-19

**Authors:** Lily Sarrafha, Gustavo M. Parfitt, Ricardo Reyes, Camille Goldman, Elena Coccia, Tatyana Kareva, Tim Ahfeldt

**Affiliations:** 1 Nash Family Department of Neuroscience at Mount Sinai, New York, NY 10029, USA; 2 Department of Neurology at Mount Sinai, New York, NY 10029, USA; 3 Department of Cell, Developmental and Regenerative Biology at Mount Sinai, New York, NY 10029, USA; 4 Ronald M. Loeb Center for Alzheimer’s Disease at Mount Sinai, New York, NY 10029, USA; 5 Friedman Brain Institute at Mount Sinai, New York, NY 10029, USA; 6 Black Family Stem Cell Institute at Mount Sinai, New York, NY 10029, USA

**Keywords:** Cell isolation, Neuroscience, Stem Cells, Cell Differentiation, Organoids

## Abstract

Here, we describe a high-throughput 3D differentiation protocol for deriving midbrain dopaminergic neurons from human pluripotent stem cells. The use of organoids has become prevalent in disease modeling, but there is a high demand for more homogeneous cultures. Our approach is advantageous for large-scale production of uniform midbrain organoids that can be maintained in diverse formats, and our reporters allow for sorting of dopaminergic neurons. The maturing long-term organoid cultures can be used as a model for the entire midbrain.

For complete details on the use and execution of this protocol, please refer to Ahfeldt et al. (2020).

## Highlights

• High-throughput generation of 3D midbrain organoids from hPSCs in spinner flasks
• Generation of reporter hPSC lines for dopaminergic neurons
• Dopaminergic neurons cultured in 2D, 2.5D, and 3D formats
• Maturation in long-term midbrain organoid cultures

## Graphical abstract

**Figure:**
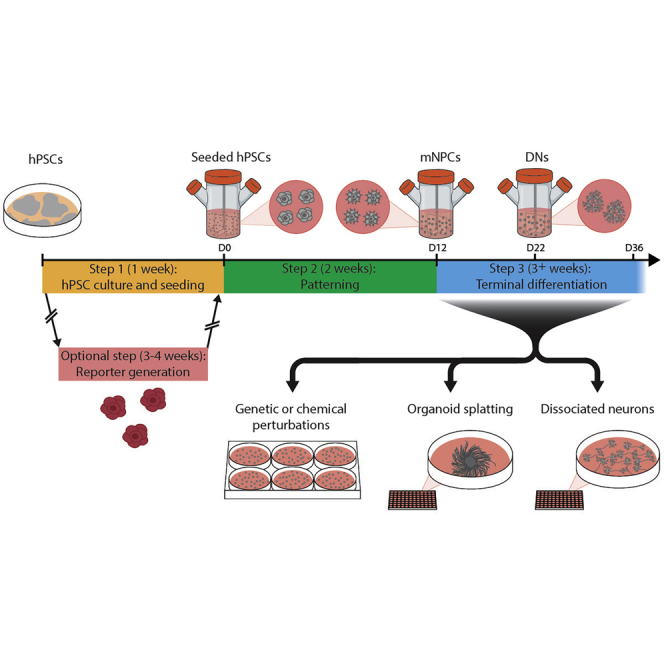

## Before you begin

Prepare the materials below for each section before starting the protocol. Refer to key resources table for a complete list of reagents and tools, and to materials and equipment for details on reagent preparation. Before you begin, review the key steps of the protocol using the flowchart in Figure S1.

***Note:*** All procedures are performed in a Class II biological hood with standard aseptic technique. Cells and midbrain organoids are cultured in a humidified 37°C incubator with 5% CO_2_.

### Generation of TH-TdTomato reporter human pluripotent stem cell (hPSC) lines (optional step)

1. Geltrex-coated 10-cm dishes (see Geltrex solution)
2. Wash buffer (see hPSC wash buffer)
3. Nucleofection mix (see nucleofection mix)
4. StemFlex medium + 2 μM Thiazovivin (pre-warmed to 37°C)
5. Microscope in tissue culture (TC) hood for colony picking
6. Geltrex-coated 12-well plates
7. StemFlex stock (see hPSC StemFlex stock)

### Seeding and maintenance of hPSC lines in spinner flasks

8. Nine-position stir plate set to 65 rpm in the incubator
9. StemFlex stock (see seeding StemFlex stock)
10. Wash buffer (see seeding wash buffer)

### Differentiation of pluripotent spheres into midbrain progenitor organoids

11. Stocks for differentiation reagents (see preparation of reagent stocks)
12. Differentiation medium (pre-warmed to 37°C, see preparation of media).
13. Poly-L-Ornithine (PLO) and laminin-coated plates (see PLO/Laminin-coated plates)
14. Organoid dissociation (see reagents and tools for dissociation)

### Maturation of midbrain progenitor organoids into dopaminergic neurons (DNs)

15. Differentiation medium (pre-warmed to 37°C, see preparation of media).

## Key resources table

**Table:** 

REAGENT or RESOURCE	SOURCE	IDENTIFIER
**Antibodies**		

Rabbit Monoclonal Anti-FOXA2 (Clone EPR4466)	Abcam	Cat#Ab108422; RRID: AB_11157157
Rabbit Polyclonal Anti-GAPDH	Abcam	Cat#Ab9485; RRID: AB_307275
Mouse Monoclonal Anti-GFAP (Clone GA5)	Millipore	Cat#MAB360; RRID: AB_11212597
Rabbit Polyclonal Anti-RFP	Rockland	Cat#600-401-379; RRID: AB_2209751
Mouse Monoclonal Anti-TH (Clone LNC1)	Millipore	Cat#MAB318; RRID: AB_2201528
Mouse Monoclonal Anti-Beta III Tubulin or TUBB3 (Clone 2G10)	Abcam	Cat#Ab78078; RRID: AB_2256751

**Chemicals, peptides, and recombinant proteins**		

Accutase® Cell Detachment Solution	Innovative Cell Technologies	Cat#AT104; RRID: AB_2869384
B-27™ Supplement (50×), Minus Vitamin A	Gibco	Cat#12587010; RRID: N/A
Bovine Albumin Fraction V (7.5% Solution)	Gibco	Cat#15260037; RRID: N/A
CHIR99021	Tocris	Cat#4423; RRID: N/A
DAPT	Cayman Chemical	Cat#13197; RRID: N/A
DB-cAMP/Dibutyryl-cAMP	Biolog	Cat#D 009; RRID: N/A
DirectPCR Lysis Reagent (Cell)	Viagen Biotech	Cat#302-C; RRID: N/A
DMEM/F-12, GlutaMAX™ Supplement	Gibco	Cat#10565018; RRID: N/A
DMEM, High Glucose	Gibco	Cat#11965092; RRID: N/A
DNase Vial (D2)	Worthington Biochemical Corporation	Cat#LK003170; RRID: N/A
Geltrex™ LDEV-Free, hESC-Qualified, Reduced Growth Factor Basement Membrane Matrix	Gibco	Cat#A1413302; RRID: N/A
HBSS with 10 mM HEPES, Without Phenol Red	STEMCELL Technologies	Cat#37150; RRID: N/A
Hibernate A	BrainBits	Cat#HA; RRID: N/A
Hoechst 33342, Trihydrochloride, Trihydrate - 10 mg/mL Solution in Water	Invitrogen	Cat#H3570; RRID: N/A
Laminin Mouse Protein, Natural	Gibco	Cat#23017015; RRID: N/A
L-Ascorbic Acid (White Crystalline Powder)	Fisher Scientific	Cat#BP351-500; RRID: N/A
LDN193189	Stemgent	Cat#04-0074; RRID: N/A
Leibovitz's L-15 Medium	Gibco	Cat#11415064; RRID: N/A
N-2 Supplement (100×)	Gibco	Cat#17502048; RRID: N/A
Opti-MEM™ I Reduced Serum Medium	Gibco	Cat#31985062; RRID: N/A
PBS, pH 7.4 (-CaCl_2_, -MgCl_2_)	Gibco	Cat#10010023; RRID: N/A
PDS Kit, Papain Vial	Worthington Biochemical Corporation	Cat#LK003176; RRID: N/A
Penicillin-Streptomycin (10,000 U/mL)	Gibco	Cat#15140122; RRID: N/A
Poly-L-Ornithine Hydrobromide	Sigma-Aldrich	Cat#P3655; RRID: N/A
Proteinase K Solution (20 mg/mL)	Viagen Biotech	Cat#501-PK; RRID: N/A
Purmorphamine	STEMCELL Technologies	Cat#72204; RRID: N/A
Puromycin Dihydrochloride	Gibco	Cat#A1113803; RRID: N/A
Recombinant Human BDNF Protein	R&D Systems	Cat#248-BD; RRID: N/A
Recombinant Human GDNF Protein	R&D Systems	Cat#212-GD; RRID: N/A
SAG	Cayman Chemical	Cat#11914; RRID: N/A
SB431542	Stemgent	Cat#04-0010-10; RRID: N/A
StemFlex™ Medium	Gibco	Cat#A3349401; RRID: N/A
Thiazovivin	Selleck Chemicals	Cat#S1459; RRID: N/A
Trypan Blue Solution, 0.4%	Gibco	Cat#15250061; RRID: N/A
Y-27632 Dihydrochloride	Tocris	Cat#1254; RRID: N/A

**Critical commercial assays**		

Fontana-Masson Stain Kit (Melanin Stain)	Abcam	Cat#Ab150669; RRID: N/A
Lipofectamine™ Stem Transfection Reagent	Invitrogen	Cat#STEM00003; RRID: N/A
P3 Primary Cell 4D-Nucleofector^TM^ X Kit S	Lonza	Cat#V4XP-3032; RRID: N/A

**Experimental models: Cell lines**		

Human: BJ SiPS-D induced pluripotent stem cells	Harvard University	Cat#BJ SiPS-D: RRID: CVCL_X741
Human: WA01 (H1) embryonic stem cells	WiCell Research Institute	Cat#WA01; RRID: CVCL_9771

**Oligonucleotides**		

Primer: TH-TdTomato Forward: AGCCCTCTAGCCTCATCCTC	This paper	RRID: N/A
Primer: TH-TdTomato Reverse: GAGCCTCTGGAGCTGCTTG	This paper	RRID: N/A
Primer: 5Prime Reverse: ACATCCCCTGCTTGTTTCAA	This paper	RRID: N/A
Primer: 3Prime post-Cre Forward: TCCCTCAGACCCTTTTAGTCA	This paper	RRID: N/A
Primer: ACTB Forward: CCTGGCACCCAGCACAAT	Pilegaard et al., 2003	RRID: N/A
Primer: ACTB Reverse: GCCGATCCACACGGAGTACT	Pilegaard et al., 2003	RRID: N/A
Primer: FOXA2 Forward: GGGGTAGTGCATCACCTGTT	Jo et al., 2016	RRID: N/A
Primer: FOXA2 Reverse: CCGTTCTCCATCAACAACCT	Jo et al., 2016	RRID: N/A
Primer: GIRK2 (KCNJ6) Forward: TGCCCAAAGAGGAACTGGAAAT	This paper	RRID: N/A
Primer: GIRK2 (KCNJ6) Reverse: AGTCAACTTCGTAGAACCCGTC	This paper	RRID: N/A
Primer: LMX1A Forward: GCAAAGGGGACTATGAGAAGGA	PrimerBank	Cat#291327512c1; RRID: N/A
Primer: LMX1A Reverse: CGTTTGGGGCGCTTATGGT	PrimerBank	Cat#291327512c1; RRID: N/A
Primer: NURR1 (NR4A2) Forward: CAGGCGTTTTCGAGGAAAT	Kirkeby et al., 2017	RRID: N/A
Primer: NURR1 (NR4A2) Reverse: GAGACGCGGAGAACTCCTAA	Kirkeby et al., 2017	RRID: N/A

**Recombinant DNA**		

Plasmid: pTAHR TH-p2a-TD:Tomato (floxed selection Puro)	Ahfeldt et al., 2020; Addgene	Cat#135814; RRID: N/A
CRISPR 1: pTACR TH1-p2aGFP	Ahfeldt et al., 2020; Addgene	Cat#135815; RRID: N/A
CRISPR 2: pTACR TH2-p2aGFP	Ahfeldt et al., 2020; Addgene	Cat#135816; RRID: N/A
Plasmid: pCAG-Cre:GFP	Matsuda and Cepko, 2007; Addgene	Cat#13776; RRID: Addgene_13776

**Software and algorithms**		

CellProfiler	https://cellprofiler.org/	RRID:SCR_007358
Fiji	https://fiji.sc/	RRID:SCR_002285

**Other**		

4D-Nucleofector^TM^ Core Unit	Lonza	Cat#AAF-1002B; RRID: N/A
Cell Culture Microplate, 96-well, PS, F-bottom (Chimney Well), μClear®, Black, CELLCOAT®, Poly-L-Lysine, Lid with Condensation Rings, 5 pcs./bag	Greiner	Cat#655936; RRID: N/A
Corning® 125 mL Disposable Spinner Flask with 70 mm Top Cap and 2 Angled Sidearms, Sterile	Corning	Cat#3152; RRID: N/A
Corning® Costar® Ultra-Low Attachment Multiple Well Plate	MilliporeSigma	Cat#CLS3471; RRID: N/A
Countess™ Cell Counting Chamber Slides	Invitrogen	Cat#C10228; RRID: SCR_019815
Countess™ II FL Automated Cell Counter	Invitrogen	Cat#AMQAF1000; RRID: N/A
Dropper Bulb, Natural Latex Rubber, 2 mL, 12 per Case	Research Products International	Cat#142823; RRID: N/A
Falcon® Round-Bottom Tubes with Cell Strainer Cap, 5 mL	STEMCELL Technologies	Cat#100-0087; RRID: N/A
Fisherbrand™ Disposable Borosilicate Glass Pasteur Pipets	Fisher Scientific	Cat#13-678-20D; RRID: N/A
Fisherbrand™ Sterile Cell Strainers (70 μm)	Fisher Scientific	Cat#22-363-548; RRID: N/A
pluriStrainer® 300 μm, 25 pcs. – Sterile (Cell Strainer)	pluriSelect	Cat#43-50300-03; RRID: N/A
pluriStrainer® 500 μm, 25 pcs. – Sterile (Cell Strainer)	pluriSelect	Cat#43-50500-03; RRID: N/A
Stirrers, Magnetic, Nine-position, Dura-Mag	Chemglass Life Sciences	Cat#CLS-4100-09; RRID: N/A
Variable Speed 2D Rocker	USA Scientific	Cat#2527-2000; RRID: N/A

## Materials and equipment

### Preparation of reagent stocks

• LDN193189: 10 mM in ddH_2_O
• SB431542: 50 mM in DMSO
• Purmorphamine: 10 mM in DMSO
• SAG: 10 mM in DMSO
• CHIR99021: 15 mM in DMSO
• BDNF: 20 μg/mL in 0.1% BSA in PBS
• GDNF: 20 μg/mL in 0.1% BSA in PBS
• Ascorbic Acid: 40 mM in ddH_2_O
• DAPT: 50 mM in DMSO
• dcAMP: 20 mM in ddH_2_O

***Note:*** Aliquot and store all reagent stock solutions at −20°C. Follow the manufacturer’s guidelines for the duration of storage for each reagent.

**CRITICAL:** It is important to limit the volume of DMSO used in reagent stocks since its recommended maximum final concentration for cell culture is 0.1% and DNs are especially sensitive to it.

**CRITICAL:** This protocol is sensitive to CHIR99021 concentration and optimization may be required for different hPSC lines. Recommended concentrations for 2D midbrain cultures range from 0.6 to 1 μM (Nolbrant et al., 2017) or as high as 3 μM CHIR99021 (Kriks et al., 2011). A concentration range of 1.5 to 3 μM has been working well for our 3D protocol in independent cell lines and we use 1.5 μM here in this study. In addition, we have observed variability in CHIR99021 activity from different vendors. Therefore, we recommend using the same vendor and purchasing this compound in bulk to avoid potential batch-to-batch variability.

### Preparation of media

**Table:** 

Medium	Components
D0-1 (Patterning)	DMEM/F-12+Glutamax + 1× B-27 minus vitamin A + 1× N-2 + 100 nM LDN193189 + 10 μM SB431542
D2-3 (Patterning)	DMEM/F-12+Glutamax + 1× B-27 minus vitamin A + 1× N-2 + 100 nM LDN193189 + 10 μM SB431542 + 2 μM Purmorphamine + 1 μM SAG
D4-7 (Patterning)	DMEM/F-12+Glutamax + 1× B-27 minus vitamin A + 1× N-2 + 100 nM LDN193189 + 10 μM SB431542 + 2 μM Purmorphamine + 1 μM SAG + 1.5 μM CHIR99021
D8-11 (Patterning)	DMEM/F-12+Glutamax + 1× B-27 minus vitamin A + 1× N-2 + 100 nM LDN193189 + 1.5 μM CHIR99021
D12-21 (Terminal)	DMEM/F-12+Glutamax + 1× B-27 minus vitamin A + 1× N-2 + 20 ng/mL BDNF + 20 ng/mL GDNF + 0.2 mM Ascorbic Acid + 10 μM DAPT + 0.1 mM dcAMP
D22-35 (Terminal)	DMEM/F-12+Glutamax + 1× B-27 minus vitamin A + 1× N-2 + 10 ng/mL BDNF + 10 ng/mL GDNF + 0.2 mM Ascorbic Acid + 10 μM DAPT + 0.1 mM dcAMP
D36^+^ (Terminal)	DMEM/F-12+Glutamax + 1× B-27 minus vitamin A + 1× N-2 + 10 ng/mL BDNF + 10 ng/mL GDNF + 0.2 mM Ascorbic Acid

***Note:*** The name of each medium denotes the days of differentiation on which it is used. Prepare appropriate medium freshly every 2 days. Store media at 4°C for a maximum of 1 week.

***Alternatives:*** The use of alternative reagents for the differentiation steps is not recommended as this protocol has been optimized for the specific reagents mentioned above. However, reagents from other published midbrain differentiation protocols may be used if proper optimization and quality control (QC) steps are followed.

### Preparation of buffers and solutions

#### Geltrex solution

•Thaw Geltrex at 4°C.
•Dilute at 1:100 in cold high glucose DMEM basal medium.
•Store at 4°C for up to 1 week.

#### hPSC wash buffer

•PBS + 2% ‘Bovine Albumin Fraction V 7.5% Solution’ (referred to as ‘BSA’ from here on) + 2 μM Thiazovivin ROCK inhibitor (final Bovine Albumin Fraction V concentration: 0.15%)
•Prepare freshly for each experiment.

#### Nucleofection mix

•Amaxa P3 Primary Cell 4D-Nucleofector Kit: 2.25 mL Nucleofector Solution + 0.5 mL Supplement 1
•Store at 4°C for up to 3 months.

#### hPSC StemFlex stock

•StemFlex medium + 2 μM Thiazovivin + 1:100 Penicillin-Streptomycin (Pen/Strep)
•Prepare freshly for each experiment.

***Alternatives:*** Depending on the hPSC culture practices in each lab, alternative reagents can be used such as Matrigel or Cultrex instead of Geltrex, collagenase or Trypsin-EDTA instead of Accutase, mTeSR medium instead of StemFlex, and Lipofectamine instead of the Nucleofector Kit.

#### Seeding StemFlex stock

•StemFlex medium + 10 μM Y-27632 + 1:100 Pen/Strep
•Prepare freshly for each experiment.

***Note:*** We use 10 μM Y-27632 instead of 2 μM Thiazovivin as ROCK inhibitor for culturing hPSCs in spinner flasks to enhance cell survival and sphere formation. The use of Pen/Strep is optional for spinner flask cultures.

#### Seeding wash buffer

•PBS + 2% BSA + 10 μM Y-27632 ROCK inhibitor
•Prepare freshly for each experiment.

#### PLO/Laminin-coated plates

•PLO solution: PLO hydrobromide + 0.15 M Borate Buffer (pH 8.4). Filter sterilize and store at 4°C. The solution can be re-used for up to 5 times within 3 months.
•L15+Bicarbonate: 12.5 mL Sodium Bicarbonate Solution (7.5 g/l) + 500 mL L15 medium. Filter sterilize and store at 4°C for up to 3 months.
•Laminin solution in L15+Bicarbonate: final concentration of 10 μg/mL. Prepare the required amount freshly right before plating.
•Coat the desired number of wells of a 96-well μClear plate with 100 μl/well PLO solution and place in the incubator for 16–24 h.
•Remove PLO solution and wash wells with 200 μl/well ddH_2_O three time (5–10 min each).
•Coat wells with 100 μl/well laminin solution and place in the incubator for 16–24 h.
•Aspirate laminin solution and proceed to plating dissociated cells or splatted organoids. No wash steps are required before plating.

**CRITICAL:** PLO hydrobromide is extremely hygroscopic. Do not attempt to weigh.

#### Reagents and tools for dissociation

•Papain solution: 1 vial papain + 1 vial DNase + 10 mL HBSS-H (pre-warmed to 37°C; prepare freshly for each experiment)
•Incubation medium: Hibernate-A medium + 1× B27 minus vitamin A + 1% BSA (pre-warmed to 37°C; prepare freshly for each experiment)
•Fire-polished Pasteur pipets: different tip sizes prepared using a Bunsen burner

#### Antibody dilutions

•Western blot
  FOXA2 1:1000
  GAPDH 1:2500
  TH 1:1000
•Immunofluorescence Staining
  Hoechst 1:5000 (nuclear dye)
  RFP 1:500
  TH 1:500
  TUBB3 1:1000
•Immunohistochemistry
  GFAP 1:10
  TH 1:500

## Step-by-step method details

### Generation of TH-TdTomato reporter hPSC lines (optional step)

**Timing: 3–4 weeks**

**Figure 1:**
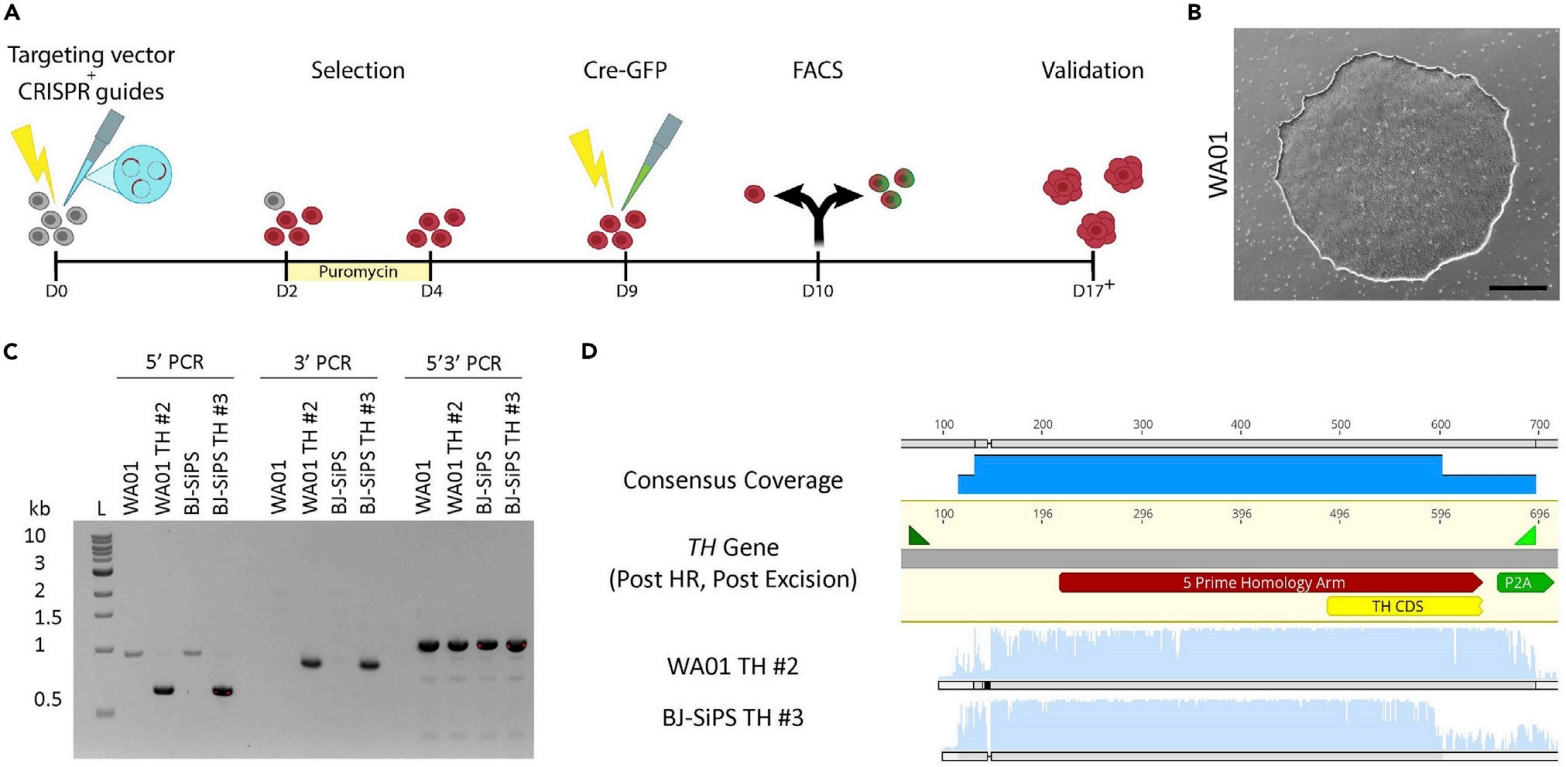
Generation and confirmation of TH-TdTomato knock-in hPSC lines (optional step) (A) Schematic overview of the CRISPR-mediated gene targeting approach. (B) Bright-field image of a WA01 colony after targeting. Scale bar: 500 μm. (C) 5**′**, 3**′**, and 5**′**3**′** PCRs confirming TH-TdTomato vector integration into selected WA01 and BJ-SiPS hPSC clones. (D) Sequencing results validating correct vector integration for two clones using the “TH-TdTomato Forward” primer for the 5**′** PCR product.

This optional section describes the targeting of our TH-TdTomato vector into hPSCs using a CRISPR-mediated knock-in strategy (Figure 1A). We use the pTAHR TH-p2a-TD:Tomato targeting vector in which the stop codon is removed to fuse the Tyrosine Hydroxylase (TH) gene to an in-frame P2A-TdTomato sequence (Ahfeldt et al., 2020). We generated CRISPR/Cas9 constructs by cloning CRISPR guide sequences into the previously published PX330 Cas9 plasmid (Ahfeldt et al., 2020; Cong et al., 2013). For removal of the selection cassette, we use the pCAG-Cre:GFP plasmid (Matsuda and Cepko, 2007) and flow sort GFP^+^ cells via fluorescence-activated cell sorting (FACS) to grow colonies. The TH-TdTomato reporter allows for monitoring and FACS of midbrain DNs throughout the differentiation and can be used for downstream applications such as plating the neurons on astrocytes or omics studies.

***Note:*** The TH-TdTomato hPSCs have weak TdTomato fluorescence only for a few days after targeting, but in Figure 1A we show these cells in red throughout the timeline to represent the cells containing the knock-in vector.

1. Coat the desired number of 10-cm dishes with 8 mL Geltrex solution and store in a CO_2_ incubator at 37°C for at least 1 h (see Geltrex solution).
  a. Coat one additional dish for nucleofection control to ensure that the nucleofection reaction has worked properly on the following day.
2. Aspirate StemFlex medium from 70%–80% confluent hPSCs in a 10-cm dish and wash with 8 mL PBS to remove debris.
3. Aspirate PBS and incubate cells with 3 mL fresh Accutase at 20°C–25°C for 5 min with occasional manual shaking. Incubation time may vary slightly between different cell lines.

***Note:*** Accutase is a gentle cell detachment solution and therefore does not need to be neutralized during passaging. However, we reduce its activity during the centrifugation step by diluting it in at least twice the volume of wash buffer.

4. In the meantime, prepare 10 mL wash buffer in a 15-mL conical tube (see hPSC wash buffer).
5. Gently pipet the cells on the dish 3–5 times using a 5-mL pipet and add to the 15-mL tube.
6. Mix the cells 3–5 times in the 15-mL tube and take a small amount for cell count.

***Note:*** During regular TC procedures such as hPSC passaging, we dissociate the cells in small clumps since hPSCs have reduced viability when suspended as single cells. However, for nucleofection we further dissociate hPSCs into single cells to ensure that we derive clonal cell populations during cell targeting. The use of Thiazovivin enhances cell survival after nucleofection.

7. Count cells using Trypan Blue solution, Chamber Slides, and a Cell Counter before centrifugation and distribute appropriate volume of the mixture containing 2 million cells to a new 15-mL tube.
  a. Collect an additional tube with 2 million cells for nucleofection control.
8. Centrifuge cells at 300 × *g* for 3 min.
9. In the meantime, combine the following in a 1.5 mL microcentrifuge tube: 5 μg TH-TdTomato vector + 1 μg CRISPR 1 + 1 μg CRISPR 2 + 100 μl nucleofection mix (see nucleofection mix).
  a. For the nucleofection control, combine: 5 μg pmaxGFP Vector (provided by the nucleofection kit) + 100 μl nucleofection mix.
10. Aspirate the supernatant from the 15-mL tube and gently resuspend the pellet in nucleofection mix + DNA using a 200-μl pipet tip.
11. Pipet the cells once gently and transfer to a cuvette provided by the nucleofection kit.
  a. Place cuvettes on ice.
12. Nucleofect using Program Ca137 on a 4D-Nucleofector Core Unit.
  a. Place cuvettes back on ice.
13. Remove Geltrex solution from 10-cm dishes and replace with 8 mL/dish StemFlex medium + 2 μM Thiazovivin (pre-warmed to 37°C). No wash step is required after Geltrex removal.
14. Gently remove the cells from the cuvette using a pipet provided by the nucleofection kit and transfer to the prepared dishes from step 13.
15. Store the plates in the incubator.
16. On day 1 post-nucleofection observe all plates under a TC microscope to ensure that the cells look healthy and that the nucleofection control group is expressing GFP.
  a. Do not change media on this day.

***Note:*** The peak of GFP expression occurs at around 16 h post-nucleofection.

17. On day 2 post-nucleofection begin selection with 1 μg/mL puromycin for 48 h to select for transgene integration. Supplement StemFlex medium with puromycin and change media daily. Thiazovivin is not added at this point.

***Note:*** It is recommended to perform a puromycin kill curve test before this step when working with new hPSC lines to determine the best working concentration (troubleshooting 1).

18. On day 4 post-nucleofection replace media with fresh StemFlex medium without puromycin.
19. Change media every other day until hPSC colonies are clearly visible by eye (5–7 days).

**Expected outcome:** 10–50 puromycin-resistant colonies per 10-cm dish 5–7 days post-selection depending on the cell line used. The hPSC colonies derived during targeting generally arise from single cells post-selection and therefore have a circular morphology. The colonies are visible by eye when they reach ~1–2 mm in diameter (Figure 1B).

20. Passage the cells as described in steps 1–15. Pool all colonies together using Accutase by additional trituration with a 1-mL pipet tip to break down chunks of cells. A longer Accutase incubation time may be required for this step.
21. Use half of the cell suspension for Cre-GFP targeting. Freeze the other half into 2 or more cryovials (depending on the pellet size) as backup before Cre-GFP nucleofection.

**Pause point:** Alternatively, all cells can be frozen at this stage as pre-Cre targeted hPSCs. While it is recommended to avoid freeze-thaw cycles during the entire cell line derivation process, backup freezes are encouraged whenever possible.

***Note:*** If the number of cells is insufficient, the pre-Cre hPSCs can be passaged regularly once to expand before Cre-GFP targeting.

22. Repeat the nucleofection process using 5 μg Cre-GFP vector to remove the puromycin selection cassette.
  a. Include a non-nucleofected negative control as well as a GFP nucleofection control for fluorescence gating during FACS.
    ***Note:*** The nucleofection conditions described here have been optimized for the WA01 and BJ-SiPS hPSC lines used in this study. Further optimization may be required when using new lines, such as adjusting the amount of DNA added to the cells.
  b. Alternatively, lipofection can be used for targeting.
    ***Optional:*** We have successfully used Lipofectamine Stem Transfection Reagent as an alternative to nucleofection for generation of knock-in hPSC lines using the following conditions. Similar to the nucleofection protocol, optimization may be required when using new hPSC lines.
    i. Passage hPSCs to a 6-well plate at 70%–80% confluency in StemFlex medium + 2 μM Thiazovivin 1 h prior to transfection.
    ii. Prepare the following mixes in separate tubes (amount per well):
      1 mL Opti-MEM + 4 μl Lipofectamine
      1 mL Opti-MEM + 4 μg Targeting vector + 1 μg CRISPR 1 + 1 μg CRISPR 2
    iii. Combine the contents of the two tubes and incubate at 20°C–25°C for 10 min.
    iv. Replace StemFlex medium with the transfection mix and place in the incubator for 2 h.
    v. Change media to fresh StemFlex medium.
    vi. For Cre-GFP transfection, use 5 μg plasmid per reaction and proceed to perform FACS on the following day.

23. On day 1 post-nucleofection, prepare cells for FACS.

***Note:*** The peak of Cre-GFP expression occurs at around 16 h post-nucleofection. The cells can be sorted up to 48 h post-nucleofection, although we usually sort on the following day.

24. Perform FACS and plate 5,000 – 10,000 GFP^+^ cells per 10-cm Geltrex-coated dish containing 8 mL StemFlex stock pre-warmed to 37°C (see hPSC StemFlex stock).
25. On day 3 post-nucleofection, replace media with fresh StemFlex medium without Thiazovivin and Pen/Strep.
26. Change media every other day until hPSC colonies are clearly visible by eye (7–10 days).

**Expected outcome:** 10–50 post-Cre colonies per 10-cm dish 7–10 days post-nucleofection depending on the cell line used (Figure 1B). Colonies can be picked more easily when they reach a diameter of at least 1–2 mm. We generally pick 24–48 colonies per line.

27. On the day of colony picking, remove any debris by changing media to StemFlex stock.
28. Prepare desired number of uncoated 12-well plates with 0.5 mL/well StemFlex stock.
29. Coat the same number of 12-well plates with Geltrex solution. Remove Geltrex solution right before plating the colonies.
30. Pick colonies under sterile TC conditions, triturate well, and plate one colony per well of a Geltrex-coated 12-well plate in StemFlex stock.
  a. For colony picking, a sterile microscope set up in a TC hood is required. If not available, a microscope can be cleaned thoroughly with 70% ethanol and then placed inside a TC hood. Pick colonies one at a time under the microscope by scraping and collecting with a 200-μl pipet tip set to 200 μl volume and transfer to the uncoated 12-well plate. Once a plate is full, triturate each colony further with a separate 1-mL pipet tip and transfer to the Geltrex-coated plate one at a time. Store the plates in the incubator.
  b. Using the same pipet tip, collect cells from each colony in PCR strips for gDNA extraction (cell suspension volume of 100–200 μl). Centrifuge cells at 300 × *g* for 3 min and resuspend the pellets in lysis buffer. Combine 1 mL DirectPCR Lysis Reagent + 25 μl Proteinase K, resuspend cell pellets in 40–60 μl of the mix depending on the pellet size, and follow the manufacturer’s guidelines.
31. Analyze transgene integration in collected gDNA samples using touchdown PCR. We use three reactions (5**′**, 3**′**, and 5**′**3**′**) to genotype and confirm the knock-in clones (Figure 1C).

**Table:** 

Reaction	Forward primer	Reverse primer	Expected band size (bp)
5**′**	TH-TdTomato Forward	5Prime Reverse	627
3**′**	3Prime post-Cre Forward	TH-TdTomato Reverse	878
5**′**3**′**	TH-TdTomato Forward	TH-TdTomato Reverse	1044

***Note:*** No large band should be observed in the 5**′**3**′** PCR as the program does not amplify long sequences. The expected 1044 bp band size is from the untargeted or wild-type (WT) allele in heterozygous clones. No bands are observed in the 5**′**3**′** PCR for homozygous clones.

**Table:** 

PCR cycling conditions			
Steps	Temperature	Time	Cycles
Initial Denaturation	98°C	30 s	1
Denaturation	98°C	10 s	12 (Reduce the annealing temperate by 1°C in every cycle to reach 60°C at the end of the stage)
Annealing	72°C	20 s	
Extension	72°C	30 s	
Denaturation	98°C	15 s	28
Annealing	60°C	15 s	
Extension	72°C	30 s	
Final Extension	72°C	2 min	1
Hold	4°C	Forever

32. Validate positive colonies that were identified by PCR using Sanger sequencing. As an example, we have shown the sequencing results for two clones, WA01 TH #2 and BJ-SiPS TH #3, using the “TH-TdTomato Forward” primer for the 5**′** PCR (Figure 1D). The results show alignment of the 5**′** PCR products to the TH gene sequence post homologous recombination (HR) and post Cre excision.
33. Expand the selected colonies, prepare several frozen stocks, and further validate the knock-in hPSC lines by karyotyping.

***Note:*** Make sure to use a different pipet tip to aspirate the medium from each well during media change to avoid cross-contamination between colonies.

**Pause point:** All cells can be frozen at this stage as post-Cre targeted hPSCs. It is recommended to freeze a few vials as backup at the beginning of the expansion phase, followed by a large stock once the hPSC lines of interest have been verified.

**CRITICAL:** It is crucial to sequence the 5**′**3**′** PCR product of the untargeted allele as CRISPR-mediated changes (indels) are common.

### Seeding and maintenance of hPSC lines in spinner flasks

**Timing: 1–2 h**

These steps describe the process of hPSC harvesting from monolayer cultures and seeding into spinner flasks to generate 3D spheres, as well as maintenance of the pluripotent spheres until the onset of neuronal differentiation.

34. Passage hPSC lines to four 10-cm dishes for seeding into spinner flasks.
  a. Follow the passaging guidelines in steps 1–15.
  b. Seed ~40 × 10^6^ cells per flask from four 10-cm dishes at near confluency.

***Note:*** For all steps of hPSC culture except for nucleofection we use small clumps of cells for passaging to enhance cell survival. Therefore, less trituration is required after incubation with Accutase.

35. Day -5: Prepare 120 mL/flask StemFlex stock (see seeding StemFlex stock).
36. Prepare spinner flasks by carefully adding 115 mL StemFlex stock from one of the side arms and placing it on the nine-position stir plate set to 65 rpm in the incubator.
  a. Make sure that the top screw is tightly closed, while the two side screws are loosened to allow for air exchange.

***Optional:*** One vial of DNase can be added per flask to reduce potential clump formation.

**Figure 2:**
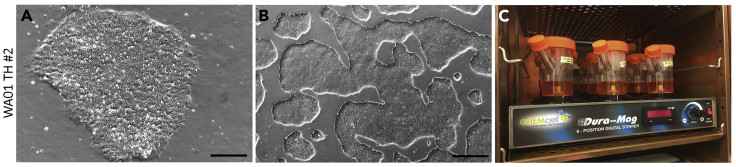
Seeding of monolayer hPSCs into 3D spinner flasks (A) Brightfield image of a healthy WA01 TH monolayer hPSC colony prior to seeding into flasks. Scale bar: 100 μm. (B) Brightfield image of WA01 TH hPSCs at 70%–80% confluency prior to seeding into flasks. Scale bar: 500 μm. (C) Image of spinner flasks on a nine-position stir plate in the incubator.
38. Aspirate StemFlex medium from the four dishes and wash with 8 mL/dish PBS.
39. Aspirate PBS and incubate cells with 3 mL/dish fresh Accutase at 20°C–25°C for 5 min with occasional manual shaking.
40. In the meantime, prepare 30 mL wash buffer (see seeding wash buffer) in a 50-mL conical tube.
41. Gently pipet the cells on each dish 3–5 times using a 5-mL pipet and add to the 50-mL tube.
42. Centrifuge cells at 300 × *g* for 3 min.
43. Aspirate the supernatant from the 50-mL tube and resuspend the pellet in 1 mL StemFlex stock using a 1-mL pipet tip.
44. Using a 5-mL pipet, add 4 mL StemFlex stock to the cells and mix.
45. Filter cells through a 35 μm cell strainer.
46. Add the cells to the flask from one of the side arms and place back in the incubator (Figure 2C).
47. Day -4: Monitor spheres daily from here on. Small spheres visible by eye are formed 24 h after seeding (troubleshooting 2).
48. Day -3: Allow the pluripotent spheres to settle by gravity, then aspirate half the media (60 mL) from one of the side arms and replace with fresh StemFlex medium + 10 μM Y-27632.
  a. If large clumps are observed, remove them by filtering spheres through a 500 μm strainer into a 50-mL tube, then gently return the spheres to the flask.
49. Day -1: Allow the pluripotent spheres to settle by gravity, then replace half the media with fresh StemFlex medium without Y-27632.
  a. Begin differentiation on D0 after filtering through a 300 μm strainer.

***Note:*** Depending on the hPSC line used a shorter or longer time may be required to reach D0.

37. Ensure that hPSCs show healthy stem cell morphology and are at 70%–80% confluency on day of seeding with merged colonies and a few remaining individual colonies (Figures 2A and 2B). Unlike the hPSC colonies derived during targeting, those in regular culture have a less circular morphology as they arise from clumps of cells rather than single cells.

### Differentiation of pluripotent spheres into midbrain progenitor organoids

**Timing: 2 weeks**

This section describes the patterning of pluripotent spheres into organoids containing midbrain neural progenitor cells (mNPCs), as well as QC methods to ensure that the patterning worked properly.

**Figure 3:**
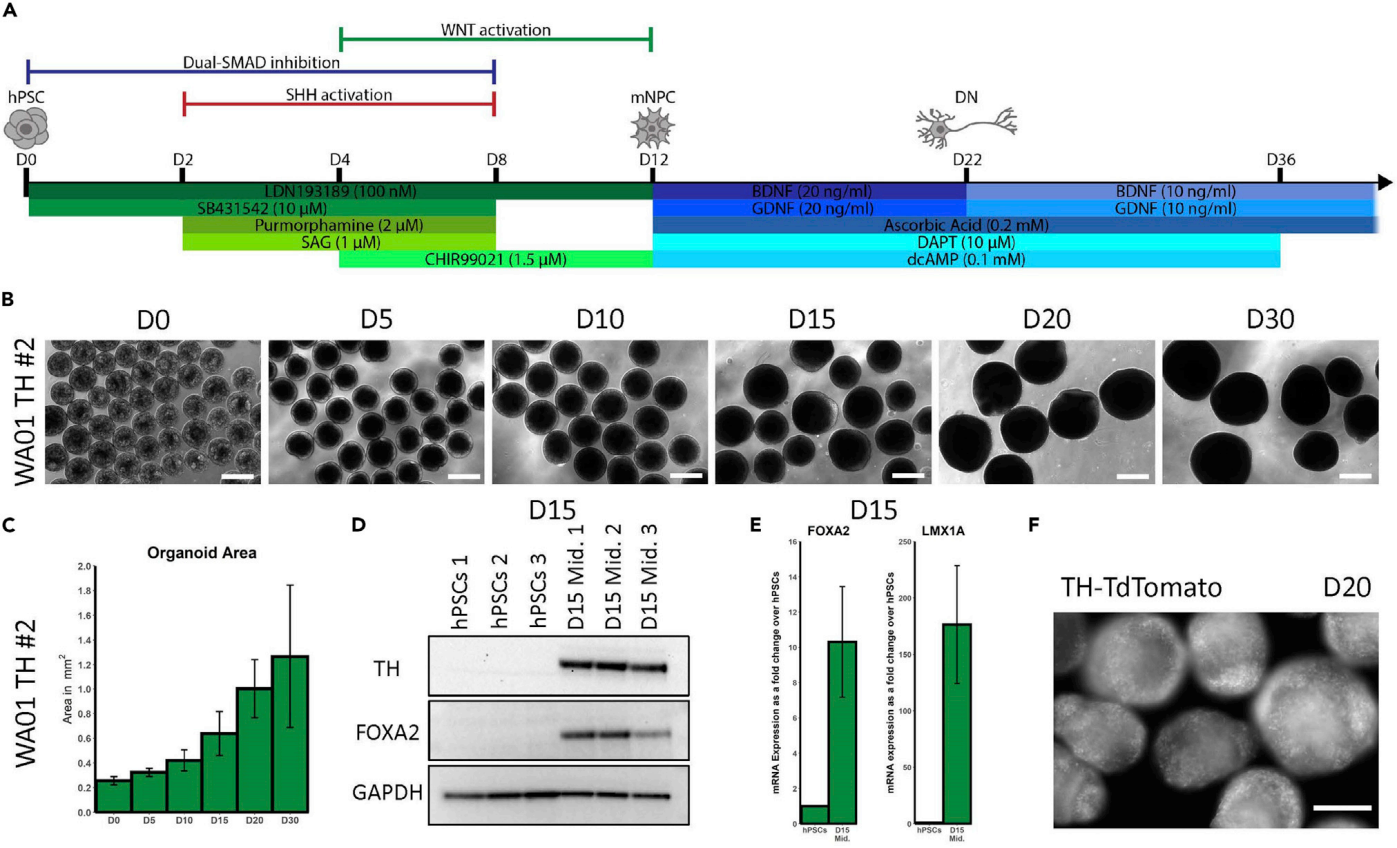
Differentiation of pluripotent spheres into midbrain progenitor organoids (A) Differentiation schematic from pluripotent spheres into mNPCs and midbrain organoids containing mature DNs. (B) Time course bright-field images of WA01 TH midbrain organoids on D0, D5, D10, D15, D20, and D30. Scale bars: 500 μm. (C) Time course quantification of the images in B showing increase in WA01 TH midbrain organoid size from D0 to D30 (n=15; 5 organoids in triplicates were pooled together for quantification). Organoid area is shown in mm^2^. Error bars represent SD. (D) WB of D15 WA01 TH midbrain organoids expressing the DN marker, TH, and midbrain progenitor marker, FOXA2. GAPDH was used as loading control. (E) qRT-PCR showing mRNA expression of midbrain progenitor markers FOXA2 and LMX1A in D15 midbrain organoids as a fold change over hPSCs (n=3). ACTB was used as a housekeeping gene for normalization. Error bars represent SD. (F) Fluorescent image of D20 WA01 TH midbrain organoids expressing the TH-TdTomato reporter. Scale bar: 250 μm.
52. Day 1: Change half the media with D0-1 patterning medium.
53. Day 2: Change half the media with D2-3 patterning medium.
54. Day 3: Change half the media with D2-3 patterning medium.
55. Day 4: Change half the media with D4-7 patterning medium.

***Note:*** CHIR99021 promotes mNPC proliferation. The organoids will begin to grow at a faster rate from here on and the media may turn more yellow orange (Figures 3B and 3C, troubleshooting 3). If too much media is being used up by the cells, excess organoids must be removed. Alternatively, the organoids can be divided into two or more flasks or transferred into larger (500-mL) flasks.

50. Day 0: Allow the pluripotent spheres to settle by gravity, then filter through a 300 μm strainer into a 50-mL tube.
51. Begin differentiation by complete media change (120 mL) to D0-1 patterning medium (Figure 3A, see preparation of media).

56. Day 5: Change half the media with D4-7 patterning medium.
  a. By this timepoint the organoids appear smooth on the surface and have a brighter periphery, indicating the emergence of neuroectoderm upon neural induction. This change in appearance has been previously reported in other brain organoid cultures (Lancaster and Knoblich, 2014).
57. Day 6: Change half the media with D4-7 patterning medium.
58. Day 7: Change half the media with D4-7 patterning medium.
59. Day 8: Change most of the media (80 mL) with D8-11 patterning medium.

***Note:*** A larger volume is changed on this day to reduce the concentrations of Purmorphamine and SAG in the flasks.

60. Day 9: Change half the media with D8-11 patterning medium.
61. Day 10: Change half the media with D8-11 patterning medium.
62. Day 11: Change half the media with D8-11 patterning medium.
63. Day 12: Change most of the media (80 mL) with D12-21 terminal medium.
  a. Continue to change media daily until D22.

***Note:*** A larger volume is changed on this day to reduce the concentration of CHIR99021 in the flasks.

***Optional:*** At this point the midbrain progenitor organoids can be transferred to low-attachment 6-well plates on a shaker if several conditions or treatments are required, for example for genetic or chemical perturbation studies.

64. Day 15^+^: Perform QC experiments on the organoids for midbrain progenitor and DN markers such as FOXA2, LMX1A, EN1, OTX2, CORIN, and TH using Western blot (WB), immunofluorescence (IF) staining, or qRT-PCR to confirm midbrain identity (Kirkeby et al., 2017). As an example, we have shown WB results for FOXA2 and TH, as well as qRT-PCR results for FOXA2 and LMX1A in D15 organoids (Figures 3D and 3E, see antibody dilutions). The qRT-PCR primers used in this study are listed in key resources table.
  a. In addition, the midbrain organoids can be dissociated using papain solution and plated as 2D monolayer cultures for live imaging or fixation for IF staining (see reagents and tools for dissociation). We recommend using 96-well μCLEAR plates coated with PLO and laminin and plating 40,000 cells per well, although this number may have to be optimized in independent hPSC lines (see PLO/Laminin-coated plates). The μCLEAR plates are thin on the bottom and work well for imaging.
    ***Optional:*** The following dissociation protocol can be used for sample preparation for both FACS and plating (see reagents and tools for dissociation).
    i. Wash ~20 large organoids (~1 mm in diameter) in 5 mL PBS in a well of a 6-well plate. More organoids can be used per well if they are smaller.
    ii. Incubate in 5 mL/well papain solution on a shaker placed in the incubator for 20–30 min.
    iii. Gently triturate the organoids with fire-polished Pasteur pipets of decreasing tip sizes attached to dropper bulbs.
    iv. Filter the mixture through a 70 μm mesh into a 50-mL tube containing 10 mL incubation medium.
    v. Centrifuge cells at 800 × *g* for 5 min.
    vi. Resuspend the pellet in desired amount of FACS buffer or differentiation medium and proceed to FACS or plating.

***Optional:*** If using a TH-TdTomato line, expression of the TH reporter starting on D15 can serve as an additional QC step (Figure 3F). In addition, organoids can be plated on PLO/laminin-coated plates starting on D15 to allow for axonal outgrowths upon organoid attachment to the well surface (see PLO/Laminin-coated plates). This approach, referred to as “organoid splatting,” is a form of 2.5D culture that allows for general observation of axonal morphology as well as TH expression in 2D DN axons in TH-TdTomato reporter lines, while preserving the 3D structure of the organoid (see step 67).

### Maturation of midbrain progenitor organoids into DNs

**Timing: 3**^**+**^**weeks**

These steps describe the maintenance and maturation of midbrain progenitor organoids into DNs, as well as QC methods for the maturing midbrain organoids.

***Note:*** The onset of TH expression may vary slightly in different hPSC lines. However, in our experience, a few cells begin showing TH around D15 followed by a spike in its expression within a few days. By D20 there should be a robust reporter expression in the organoids and TH-TdTomato^+^ neurons are clearly visible under the microscope (Figure 3F).

**Figure 4:**
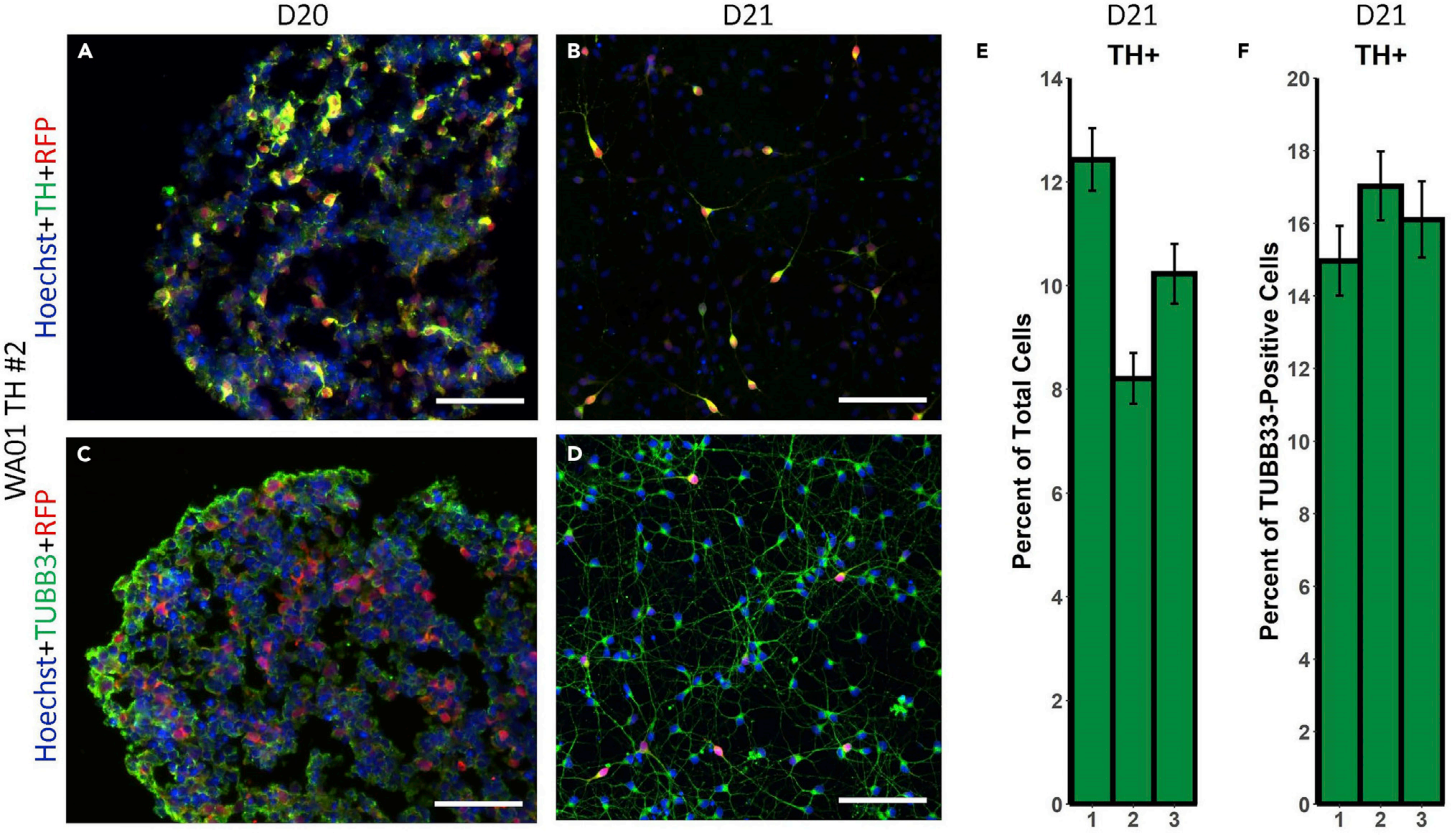
QC immunostaining of young midbrain organoids for neuronal and DN markers (A and B) IF staining of WA01 TH midbrain neurons for TH (green) and RFP (red) in (a) cryosection of a D20 organoid; (b) D21 dissociated neurons. The nuclei are counterstained with Hoechst (blue). (C and D) IF staining of WA01 TH midbrain neurons for TUBB3 (green) and RFP (red) in (c) cryosection of a D20 organoid; (d) D21 dissociated neurons. The nuclei are counterstained with Hoechst (blue). (E) Quantification of D21 TH^+^ cells as a percentage of total cells in B and similar images (n=50 per replicate). Error bars represent SEM. (F) Quantification of D21 TH^+^ cells (as denoted by RFP staining for the TH-TdTomato reporter) as a percentage of TUBB3^+^ cells in D and similar images (n=35 per replicate). Error bars represent SEM. All scale bars: 100 μm.
66. Day 22: Switch to D22-36 terminal medium (see preparation of media).
  a. From here on the media can be changed every other day if the rate of nutrient uptake is slowing down. However, daily media changes must be maintained if the color is still turning yellow orange.
  b. It is recommended to plate D22^+^ dissociated neurons on human astrocytes to enhance survival. If using the TH-TdTomato reporter line, DNs can be sorted and plated in mixed cultures or used for other downstream applications. The scalability of our protocol allows for a large number of DNs to be sorted at a time, although this step is easier and faster at later timepoints when TH expression has increased.

***Note:*** The concentrations of BDNF and GDNF are reduced at this point as these reagents have been shown to mask DN-specific phenotypes *in vitro* (Stahl et al., 2011).

**Figure 5:**
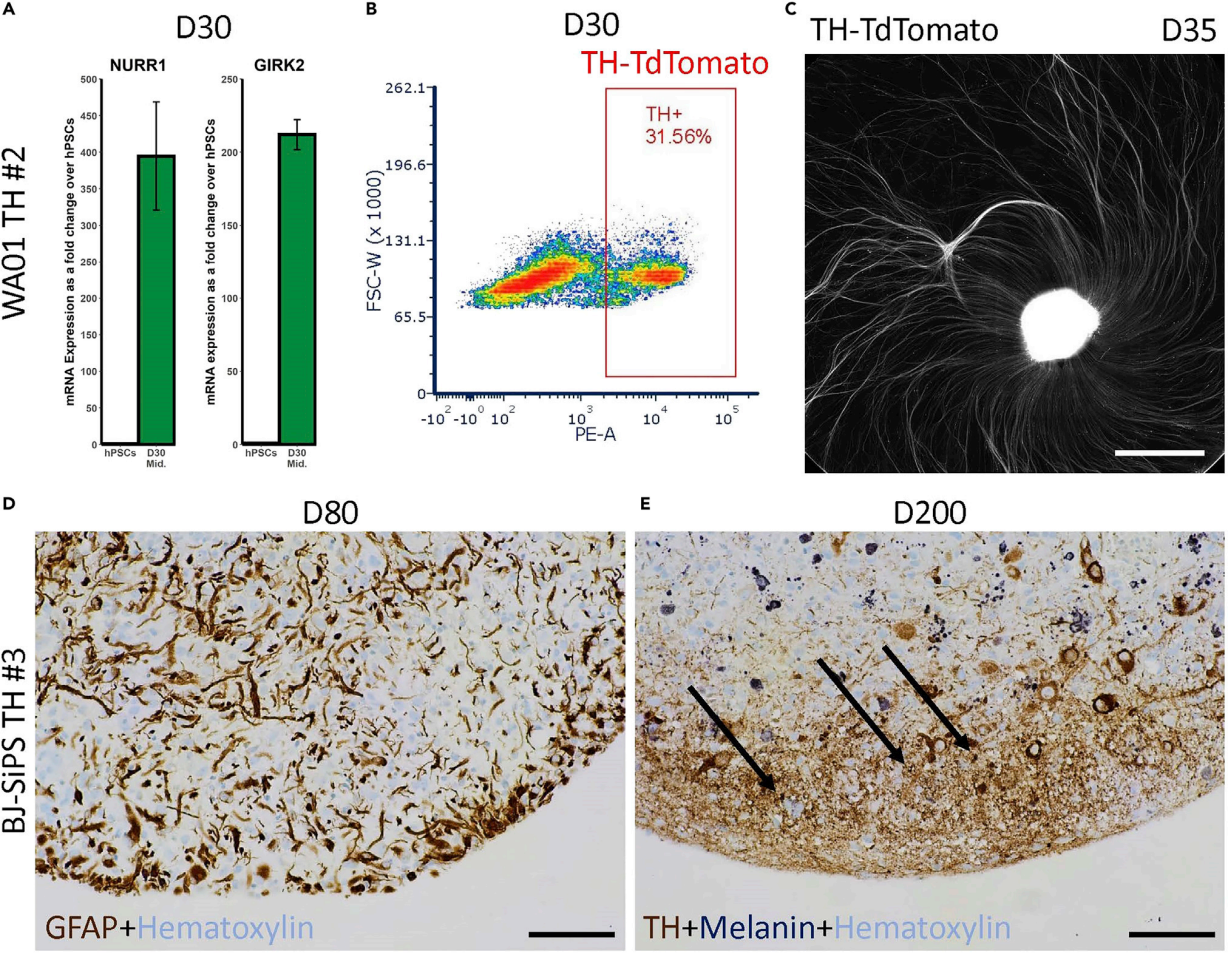
Long-term culture of mature midbrain organoids (A) qRT-PCR showing mRNA expression of mature midbrain DN markers NURR1 and GIRK2 in D30 midbrain organoids as a fold change over hPSCs (n=3). ACTB was used as a housekeeping gene for normalization. Error bars represent SD. (B) FACS analysis of dissociated D30 WA01 TH midbrain organoids showing a ~30% TH-TdTomato^+^ cell population. (C) Tiled images showing TH-TdTomato reporter expression in a D35 WA01 TH midbrain organoid splatted on D16. Scale bar: 1 mm. (D) IHC staining of a D80 BJ-SiPS TH midbrain organoid section indicating the presence of astrocytes via GFAP (brown) expression. The nuclei are counterstained with hematoxylin (light blue). Scale bar: 100 μm. (E) IHC staining of a D200 BJ-SiPS TH midbrain organoid section showing TH (brown) and melanin (dark blue) expression. Arrows point to examples of regions in which melanin is localized to DNs. The nuclei are counterstained with hematoxylin (light blue). Scale bar: 100 μm.

***Note:*** DAPT is removed at this point since it has been shown to inhibit astrocyte proliferation and may influence phenotypes associated with mature DNs (Roberts et al., 2017; Zhang et al., 2015). Therefore, switching to D36^+^ terminal medium may cause further growth in organoid size over time. Similarly, dcAMP is removed in D36^+^ medium as it has a neuroprotective effect on midbrain DNs (Momčilović et al., 2014) and may therefore have an impact on *in vitro* DN-related phenotypes.

65. Day 20^+^: From this early timepoint, the expression of TH, as well as neuronal markers such as TUBB3, can be monitored by IF staining in fixed organoid cryosections (Figures 4A and 4C) or dissociated neurons (Figures 4B and 4D, see antibody dilutions). TH^+^ cells in a monolayer culture of dissociated neurons were quantified using the Fiji and CellProfiler software for neurons stained with antibodies against TH and RFP (for the TH-TdTomato reporter). TH^+^ cells comprise approximately 10% of the total population and 16% of the TUBB3^+^ neuronal population on D21 (Figures 4E and 4F). These values may be variable between cell lines, but TH expression is expected to continue to increase until DN maturation around D36. For a kinetic analysis of TH expression using our reporter system please refer to Ahfeldt et al. (2020).

67. Day 36: Switch to D36^+^ terminal medium.
  a. The organoids are ~1 mm in diameter by this timepoint (Figure 3B).
  b. For long-term culture, media can be changed once every 3–4 days if the color is not turning yellow orange and if the organoids are not too crowded.
  c. Perform QC experiments on the organoids for mature DN markers such as NURR1 (NR4A2), GIRK2 (KCNJ6), PITX3, DAT1 (SCL6A3), VMAT2 (SLC18A2), DDC, and TH to confirm their identity (Hartfield et al., 2014). As an example, we have shown qRT-PCR results for NURR1 and GIRK2 in D30 organoids (Figure 5A).

***Optional:*** If using a TH-TdTomato line, the increase in TH expression over time can be observed in mature DNs. The midbrain organoids can also be dissociated and analyzed by flow cytometry or sorted for downstream applications such as plating on astrocytes or bulk transcriptomics and other omics studies. Flow cytometry analysis of dissociated WA01 TH organoids shows a 30% TH-TdTomato^+^ cell population on D30 of differentiation (Figure 5B, troubleshooting 4). An advantage of this approach over fluorescent imaging is that the cells that express lower levels of TH can be detected more easily as the entire TH-TdTomato^+^ population can be visualized. Another use of our DN reporter is to splat organoids to observe axonal outgrowth in mature DNs (Figure 5C), as mentioned in step 64.

68. Long-term culture: The midbrain organoids can be cultured in flasks or on low-attachment plates on a shaker for a long period of time.
  a. Previous studies have shown the emergence of astrocytes in midbrain organoids around D60 of differentiation (Monzel et al., 2017). Immunohistochemistry (IHC) staining of D80 midbrain organoid sections shows expression of the astrocyte marker, GFAP, suggesting that long-term culture using our protocol results in astrocyte development (Figure 5D, see antibody dilutions).
  b. Neuromelanin expression is a key feature of the subpopulation of DNs located in the substantia nigra pars compacta (Zecca et al., 2001). Fontana-Masson staining of D200 midbrain organoids shows melanin expression in DNs, as indicated by TH staining, as well as in other cell types (Figure 5E), suggesting that the cultures become more mature over time and obtain characteristics of the midbrain *in vivo*, including the appearance of neuromelanin.

## Expected outcomes

The targeting of hPSCs with the TH-TdTomato knock-in vector is expected to result in 10–50 colonies after each transfection step. After the Cre-GFP step, most colonies show proper 5**′** and 3**′** integration of the targeting vector based on PCR results, showing 627 bp and 878 bp bands, respectively (Figure 1C). The 5**′**3**′** PCR does not detect the >3 kb knock-in construct as the PCR program does not amplify long sequences. However, if only one chromosome is targeted, an expected band size of 1044 bp will represent the WT allele. If the knock-in occurs in both alleles, there should be no band in the 5**′**3**′** PCR. In our experience we have only obtained heterozygous knock-in clones using the TH-TdTomato vector but sequencing of the 5**′**3**′** PCR product has shown that a considerable number of the selected clones have an alteration in the second allele. Therefore, caution must be taken to select clones that have an unchanged second allele to avoid off-target effects.

Midbrain organoids gradually increase in size from D0, when they are filtered using a 300 μm strainer, to 500 μm after the patterning stage, and reach about 1 mm in diameter by D36 (Figures 3B and 3C, troubleshooting 5). By D5 the organoids have a dark core but appear smooth on the surface and have a bright and rather translucent periphery, indicating the emergence of neuroectoderm. Upon maturation the entire organoid becomes opaque in appearance. The expression of midbrain progenitor markers, as well as the DN marker TH, can be observed at the mNPC stage on D15 (Figures 3D and 3E). The onset of TH expression may be slightly variable between different hPSC lines but should begin in a few cells around D15 and spike over the next few days. By D20 TH-TdTomato reporter expression should be clearly visible throughout the organoid (Figure 3F). The expression of mature DN markers can be monitored in midbrain organoids starting around D30 (Figure 5A). TH expression continues to increase as the DNs mature and plateaus at about 30% between D30 and D40 (Figure 5B), although there may be slight variation in differentiation efficiency between different hPSC lines.

Long-term culture of our midbrain organoids results in the appearance of cell types other than DNs, such as astrocytes, as marked by GFAP expression (Figure 5D). We also observed the emergence of melanin in our older cultures. The dark pigmentation in the organoids was clearly visible by eye and we used Fontana-Masson staining to show melanin expression in different regions of the organoid, including in DNs as marked by IHC staining for TH (Figure 5E). We have cultured our midbrain organoids up to D200 without any significant hypoxia or necrosis in the core, and longer culture times are likely to be as successful, especially since DNs are generally congregated on the organoid surface where there is more access to nutrients and oxygen. In addition, other published midbrain differentiation protocols have shown successful cryopreservation of DNs at different stages of differentiation (Drummond et al., 2020; Gantner et al., 2020; Wakeman et al., 2017). Although not yet tested with our protocol, we expect that neurons from dissociated midbrain organoids can be cryopreserved for future use.

Midbrain DNs have a characteristic pacemaking activity that can be easily discerned using electrophysiological analysis (Guzman et al., 2009). The functionality and activity of the mature DNs in our midbrain organoids can be tested by electrophysiological approaches such as patch clamp, or by other relevant methods including calcium imaging (Ahfeldt et al., 2020). In addition, dopamine release can be measured using high-performance liquid chromatography (HPLC) or a Dopamine ELISA Kit (Burbulla et al., 2017; Jalewa et al., 2017; Liste et al., 2004). Several studies have shown that midbrain DNs derived *in vitro* can successfully engraft in the brains of animal models of Parkinson’s Disease (PD) (Kriks et al., 2011; Myung et al., 2008; Wang et al., 2015). The scalability and relative uniformity of our cultures provide advantages for this protocol to be used in engraftment studies for disease modeling.

There is a high demand for an *in vitro* midbrain organoid differentiation protocol to study PD and other neurodegenerative disorders in which the midbrain is affected. Several groups have developed and optimized midbrain differentiation protocols using different approaches (Jo et al., 2016; Monzel et al., 2017; Qian et al., 2018). Our protocol advances these efforts by using a scalable 3D system which results in relatively uniform organoids. The versatility of this protocol allows for different applications including low-attachment plates for genetic or chemical perturbation studies, organoid splatting or 2.5D culture for examining axonal morphology, and 2D monolayer DN cultures or co-cultures. Our TH-TdTomato reporter for DNs allows for the monitoring of these cells throughout differentiation with downstream applications that require cell isolation. The long-term cultures and increased complexity of the organoids derived using this protocol may open new opportunities to study the midbrain and gain insight into its diverse cell types and molecular pathways.

## Limitations

This protocol is limited to nine spinner flasks per stir plate, and the maintenance of the cultures is costly due to the large volume of media used. However, the organoids can be transferred to low-attachment 6-well plates on a shaker after the patterning step to overcome these limitations. Moreover, obtaining mature DNs of age D30-40 takes a long time and even longer culture periods are required for the development of other cell types in the midbrain organoids. Planning several experiments a few days or weeks apart ensures that samples are available for experiments regularly.

We have successfully used this protocol in three independent hPSC lines, the male WA01 and BJ-SiPS lines that are shown in this study, as well as the female HUES1 line which was used in our previous work (Ahfeldt et al., 2020). However, it is important to note that variations between cell lines may be observed in the number of colonies obtained during the optional gene targeting step, as well as in the onset of TH expression and DN efficiency upon differentiation. More importantly, the protocol is sensitive to CHIR99021 concentration and it is recommended to test new cell lines with concentrations ranging from 1.5 to 3 μM for this reagent to ensure that midbrain DNs are derived efficiently.

## Troubleshooting

### Problem 1

Very few colonies grow after targeting hPSCs with the knock-in vector.

### Potential solution

The nucleofection conditions in this protocol were optimized for the hPSCs used in this study and further optimization may be required for new lines. Whether nucleofection or another transfection approach such as lipofection is used, the amount of targeting vector and CRISPR DNA may have to be optimized. We generally use 5–8 μg total DNA per nucleofection reaction, but if there is too much cell death on the following day, the amount can be reduced. In addition, if there is a considerable number of cells on the day post-nucleofection but not many colonies remain after puromycin selection, the recommended puromycin concentration of 1 μg/mL may be too high. It is crucial to perform a kill curve test for this antibiotic in untargeted hPSCs when using new lines.

### Problem 2

Very few or no small spheres emerge 24 h after seeding hPSCs into spinner flasks.

### Potential solution

There are several factors that affect sphere formation that need to be optimized for new hPSC lines. Ensure that the hPSCs look healthy and are not over 80% confluent before harvesting them for seeding. Use fresh StemFlex medium, Y-27632, and Accutase. The optional addition of DNase or skipping the filtering step before seeding into flasks may enhance sphere formation. The speed of the stir plate is another factor that can affect sphere formation, especially if there is a difference in proliferation rates of the hPSC lines. Although the organoids in this protocol were derived at a speed of 65 rpm, in the past we have used a range of 55–65 rpm depending on the lab setting or hPSC line.

### Problem 3

Midbrain progenitor organoids grow too large too quickly during the patterning stage.

### Potential solution

The D4-7 and D8-11 patterning media contain CHIR99021 which promotes cell proliferation. After the patterning stage the organoids are generally about 500 μm in diameter and continue to enlarge up to 1 mm until maturation. In our experience hPSC lines may respond differently to higher CHIR99021 concentrations, and organoids that grow too large may not differentiate efficiently. It is therefore crucial to optimize CHIR99021 concentrations for new hPSC lines.

### Problem 4

Differentiation efficiency is low based on TH expression.

### Potential solution

DNs are sensitive to environmental factors such as media conditions, and lack of nutrients can lead to reduced differentiation efficiency. It is important to change media daily during the patterning stage. If the media is turning yellow orange, especially when CHIR99021 is being used, remove excess organoids. It is best to avoid increasing the volume of media change during patterning to avoid an imbalance of small molecules in the flask. CHIR99021 controls caudalization during patterning and therefore its concentration must be optimized for new hPSC lines to ensure that the organoids reach a midbrain fate rather than forebrain or hindbrain (Nolbrant et al., 2017).

### Problem 5

Organoids have a high variability in size.

### Potential solution

In our experience, organoids that are cultured in flasks past D12 are more uniform in size compared to those that are transferred to low-attachment plates after the patterning stage. However, using plates is advantageous when several conditions or treatments are required, or when lower volumes of media are to be used. In these cases, organoid size can be controlled by adding extra filtering steps if needed. The Cell Separation Company, pluriSelect, offers a variety of strainers with different sizes up to 1 mm in mesh size that can be used for filtering organoids that are too small or large. Mature midbrain organoids from this protocol generally stay around 1 mm in diameter in long-term cultures, although the removal of DAPT starting on D36 may promote further gradual proliferation in some hPSC lines.

## Resource availability

### Lead contact

Further information and requests for resources and reagents should be directed to and will be fulfilled by the lead contact, Tim Ahfeldt (tim.ahfeldt@mssm.edu).

### Materials availability

This study did not generate new unique reagents or cell lines.

### Data and code availability

This study did not generate a new dataset.

## Supplemental information

Figure S1. Flowchart of the key steps in this protocol Schematic overview of the midbrain organoid differentiation protocol starting from hPSC culture and the optional reporter generation steps to differentiation toward long-term cultures.
