# Supplementary figures and images for: High-throughput generation of midbrain dopaminergic neuron organoids from reporter human pluripotent stem cells
Source: STAR Protoc. 2021 Apr 19;2(2):100463. doi: 10.1016/j.xpro.2021.100463 (PMC8086141; doi:10.1016/j.xpro.2021.100463)

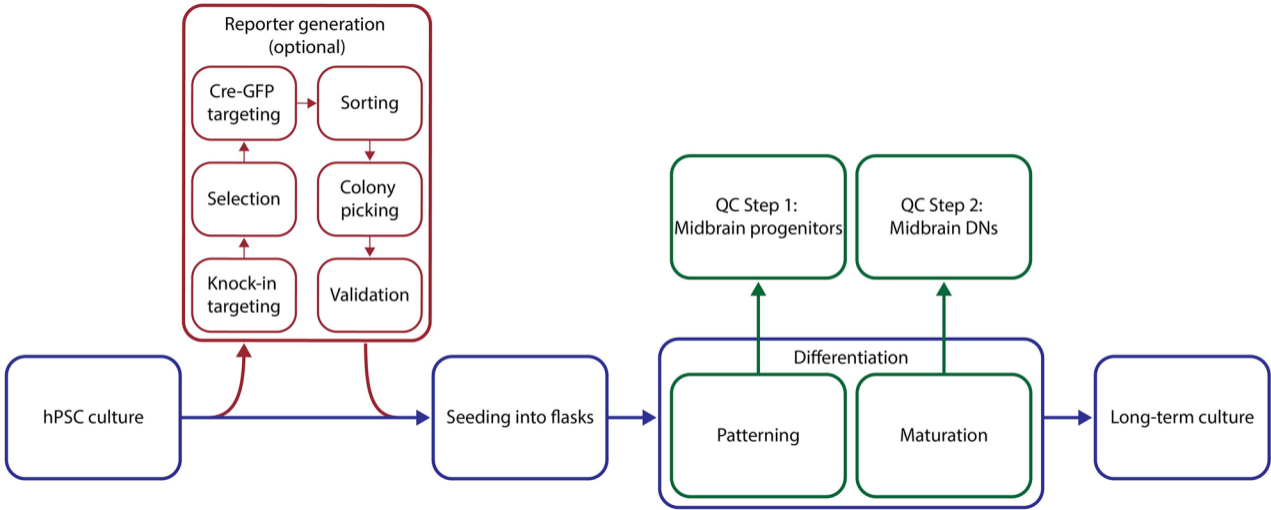

Supplement: Figure S1. Flowchart of the key steps in this protocol — Schematic overview of the midbrain organoid differentiation protocol starting from hPSC culture and the optional reporter generation steps to differentiation toward long-term cultures. [file mmc1.pdf]
